# TRPV4 Mediates Acute Bladder Responses to Bacterial Lipopolysaccharides

**DOI:** 10.3389/fimmu.2020.00799

**Published:** 2020-05-06

**Authors:** Yeranddy A. Alpizar, Pieter Uvin, Robbe Naert, Jan Franken, Silvia Pinto, Alicia Sanchez, Thomas Gevaert, Wouter Everaerts, Thomas Voets, Dirk De Ridder, Karel Talavera

**Affiliations:** ^1^Laboratory for Ion Channel Research, Department of Cellular and Molecular Medicine, VIB Center for Brain & Disease Research, Leuven, Belgium; ^2^VIB Center for Brain & Disease Research, Leuven, Belgium; ^3^Laboratory of Organ System, Department of Development and Regeneration, KU Leuven, Leuven, Belgium

**Keywords:** LPS, TRPV4, bladder, urothelial cells, cystitis

## Abstract

Urinary tract infections (UTI) affect a large proportion of the population, causing among other symptoms, more frequent and urgent micturition. Previous studies reported that the gram-negative bacterial wall component lipopolysaccharides (LPS) trigger acute epithelial and bladder voiding responses, but the underlying mechanisms remain unknown. The cation channel TRPV4 is implicated in the regulation of the bladder voiding. Since TRPV4 is activated by LPS in airway epithelial cells, we sought to determine whether this channel plays a role in LPS-induced responses in urothelial cells (UCs). We found that human-derived UCs display a fast increase in intracellular Ca^2+^ concentration upon acute application of *Escherichia coli* LPS. Such responses were detected also in freshly isolated mouse UCs, and found to be dependent on TRPV4, but not to require the canonical TLR4 signaling pathway of LPS detection. Confocal microscopy experiments revealed that TRPV4 is dispensable for LPS-induced nuclear translocation of NF-κB in mouse UCs. On the other hand, quantitative RT PCR determinations showed an enhanced LPS-induced production of proinflammatory cytokines in TRPV4-deficient UCs. Cystometry experiments in anesthetized wild type mice revealed that acute intravesical instillation of LPS rapidly increases voiding frequency. This effect was not observed in TRPV4-deficient animals, but was largely preserved in *Tlr4* KO and *Trpa1* KO mice. Our results suggest that activation of TRPV4 by LPS in UCs regulates the proinflammatory response and contributes to LPS-induced increase in voiding frequency. These findings further support the concept that TRP channels are sensors of LPS, mediating fast innate immunity mechanisms against gram-negative bacteria.

## Introduction

Epithelial cells lining the bladder and upper urinary tracts play key roles in defensive mechanisms against urinary tract infections (UTI). In addition to serving as physical barrier, urothelial cells (UCs) act as first responders upon recognition of several virulence factors, such as hemolysin and cytotoxic necrotizing factor, the siderophore aerobactin, bacterial capsules and lipopolysaccharide (LPS) (1). UCs host several defensive responses against invading bacteria, of which the most common is uropathogenic *Escherichia coli* (2, 3). These include the production of specific soluble epithelial cell-derived mediators (i.e.: lactoferrin, soluble IgA, Tamm-Horsfall protein, lipocalin), bactericidal antimocrobial peptides (defensins, cathelicidin), and bacterial clearance by urothelium exfoliation mechanisms (3). In addition, LPS recognition by Toll-like receptor 4 (TLR4) activates the transcriptional factor NF-κB, which regulates the expression of several immunomodulatory cytokines (4), causing the infiltration of inflammatory cells, and eventually edema and hemorrhage (5–8). These histological changes in the bladder wall are accompanied by burning sensation during urination, low-abdominal pain and frequent urge to urinate, typical symptoms of UTI (9).

Interestingly, there is evidence for rather fast bladder responses to LPS. For instance, acute application of LPS induces an increase of intracellular Ca^2+^ concentration within minutes and secretion of the proinflammatory cytokine IL-6 in a grade II human carcinoma bladder epithelium cell line (10). Furthermore, an increased mouse bladder voiding frequency was observed 1 h after direct intravesical instillation of LPS, that is, sooner than any histological inflammatory changes have been detected (11). Although these effects may be regarded as early bladder defensive responses against bacterial infection based LPS detection, the underlying mechanisms remain unknown.

We recently showed that airway epithelial cells respond to LPS with an elevation of cytosolic Ca^2+^ via the activation of the Transient Receptor Potential Vanilloid 4 (TRPV4) cation channel. This triggers protective responses such as production of bactericidal nitric oxide and increased ciliary beat frequency within a few minutes (12). Similar to airway epithelial cells, UCs have a prominent expression of TRPV4, and activation of this channel has been implicated in the mechanisms of bladder voiding (13, 14).

Thus, in this study we tested the hypothesis that LPS activates TRPV4 in UCs, which might lead to immediate increase in intracellular Ca^2+^ concentration, regulation of cytokine production and to changes in the bladder voiding pattern. To do this, we performed intracellular Ca^2+^ imaging experiments and NF-κB nuclear translocation and cytokine expression determinations in freshly isolated UCs. We found that *E. coli* LPS activates TRPV4 in UCs independent of the TLR4 signaling pathway. Activation of TRPV4 did not have an impact in NF-κB nuclear translocation, but we obtained evidence for a regulatory role of TRPV4 on the increase in proinflammatory cytokine expression induced by LPS. Finally, using cystometry in anesthetized mice, we found that TRPV4, but not TLR4, is required for a fast increase in voiding frequency triggered by intravesical instillation of LPS.

## Materials and Methods

### Animals

*Trpv4* KO mice were kindly provided by Prof W. Liedtke (Duke University, Durham, NC). *Tlr4* KO (B6.B10ScN-Tlr4lps-del/JthJ) mice were purchased at Charles River Laboratories (Chatillon-sur-Chalaronne, France). These knockout strains were backcrossed at least ten times into the C57BL/6J background, and C57BL/6J mice were used as wild type controls. Mice of all genotypes were housed under identical conditions, with a maximum of four animals per cage on a 12-h light-dark cycle and with food and water *ad libitum*. Ten- to twelve-week-old female mice were used in all experiments. All animal experiments were carried out in accordance with the European Union Community Council guidelines and were approved by the local ethics committee.

### Primary Urothelial Cells Culture

Isolation and culture of mouse urothelial cells was performed as previously described by others (15). After euthanasia, bladders were quickly removed, cut open, and stretched out on a Sylgard-coated dish containing MEM (Invitrogen, Merelbeke, Belgium) with 2.5 mg/ml dispase (Invitrogen) for 2 h at room temperature. After incubation, the urothelium was gently scraped from the underlying tissue, treated with trypsin-EDTA (Invitrogen) for 15 min, and resuspended in defined keratinocyte serum-free medium (Invitrogen). The cell suspension was plated on collagen (type IV; Sigma, Overijse, Belgium)-coated coverslips. Cells were used for experiments 16 h after isolation.

Human urothelial cells were collected from bladder cancer patients undergoing cystectomy, as previously described (16). Briefly, healthy bladder strips were incubated overnight at 4°C in sterile HBSS-based stripping solution containing 14 mM HEPES, 20 KIU aprotinin, 0.1% EDTA, pH 7.6. After incubation, the urothelium was gently scraped, treated with collagenase for 15 min, and resuspended in keratinocyte medium (Invitrogen). Cells were seeded in gelatin-coated glass coverslip and used for experiments 16 h after isolation.

### Intracellular Ca^2+^ Imaging

Urothelial cells were incubated with Fura-2 acetoxymethyl (2 μM) ester for 30 min at 37°C. During recordings cells were perfused by gravity via a multi-barreled pipette tip with bath solutions prepared in Krebs, containing (in mM): 150 NaCl, 6 KCl, 1.5 CaCl_2_, 1 MgCl_2_, and 10 HEPES, 10 glucose and titrated to 7.4 with NaOH. Intracellular Ca^2+^ concentration was monitored through the ratio of fluorescence measured upon alternating illumination at 340 and 380 nm using an MT-10 illumination system and the Xcellence pro software (Olympus Belgium N.V., Berchem, Belgium).

### Quantitative Real-Time PCR

Total RNA from cultured urothelial cells was extracted using the RNeasy Mini Kit (Qiagen, Antwerp, Belgium), following manufacturer’s protocol. RNA concentration was determined in a micro-volume spectrophotometer DropSense16 (Trinean NV, Gent, Belgium). cDNA synthesis was performed with 1μg of total RNA using the Ready-To-Go You-Prime First-Strand Beads (GE Healthcare, Diegem, Belgium). Quantitative PCR reactions (20 μl), containing 3 μl cDNA template (diluted 1:5), Universal TaqMan MasterMix (2x concentrated, Life Technologies), specific TaqMan probes (Table 1, 20× concentrated, Life Technologies) and H_2_O, were performed with the 7500 Fast Real-Time PCR System (Life Technologies). Reactions were made using the following program: 50°C for 2 min and 95°C for 10 min, followed by 40 cycles of 95°C for 15 s and 60°C for 1 min. Non-template controls (NTCs) were used as negative controls in every experiment.

**TABLE 1:** List of TaqMan probes.

**Gene name**	**Probe ID (in Applied Biosystems)**
*Trpv2*	Mm00449223_m1
*Trpv4*	Mm00499025_m1
*Trpm7*	Mm00457998_m1
*Tlr4*	Mm00445273_m1
*Gapdh*	Mm99999915_g1
*Il-1b*	Mm00434228_m1
*Cxcl-1*	Mm04207460_m1
*Cxcl-2*	Mm00436450_m1
*Tnf*	Mm00443258_m1

### Confocal Microscopy

Freshly isolated urothelial cells from wild type, *Trpv4* KO and *Tlr4* KO mice were seeded in glass coverslips and exposed to LPS (20 μg/ml) for 30 min. After treatment, cells were fixed with cold paraformaldehyde and permeabilized with 0.2% Triton X-100. Primary antibody against p65 NF-κB (1:250; Cell Signaling #4764) or S534-phosphorylated p65 NF-κB (1:800; Cell Signaling #3033) was incubated overnight at 4°C, followed by anti-rabbit Alexa Fluor 633 (1:600; A21070, Invitrogen) for 1 h at room temperature. Coverslips were mounted in glass slides using DAPI-containing mounting solution (VectaShield, Vector Laboratories, Burlingame, CA, United States). Confocal images were obtained from ten randomly selected fields from three independent experiments using the optimal pinhole size for the 63X oil objective on a Zeiss LSM 880-Airyscan (Carl Zeiss AG, Oberkochen, Germany). Images were analyzed using Fiji software (17) as described before (18).

### Cystometry

Catheter implantation and intravesical pressure recordings were performed as previously described (19). All recordings were performed under urethane anesthesia, and body temperature was maintained at 37°C using a heating lamp and an animal temperature controller (World Precision Instruments, Hertfordshire, United Kingdom). Bladders were infused with saline at a constant rate (20 μl/min), inducing repetitive cycles of bladder filling and voiding. After an equilibration period of 60 min, baseline intravesical pressures were recorded for 30 min. The infusion fluid was then switched to a solution containing LPS in saline, and intravesical pressures were measured again for 30 min. In a group of animals HC-067047 (1 ml at 100 μM = 2.35 mg/kg) was administered intraperitoneally 30 min prior recordings.

### Statistics

Magnitudes were statistically compared using GraphPad Prism version 7.0d for MacOS, GraphPad Software, La Jolla, CA, United States^1^ (^1^www.graphpad.com). The non-parametric Wilcoxon and Mann-Whitney *U* tests were used to determine statistically significant changes and differences in medians, respectively. Differences were considered to be statistically significant when *P* < 0.05 (labeled as ^∗^ in the Figures), but *P* < 0.01 (^∗∗^) was also indicated when applicable.

## Results

### TRPV4 Mediates LPS-Induced Ca^2+^ Entry in Freshly Isolated Urothelial Cells

**FIGURE 1:**
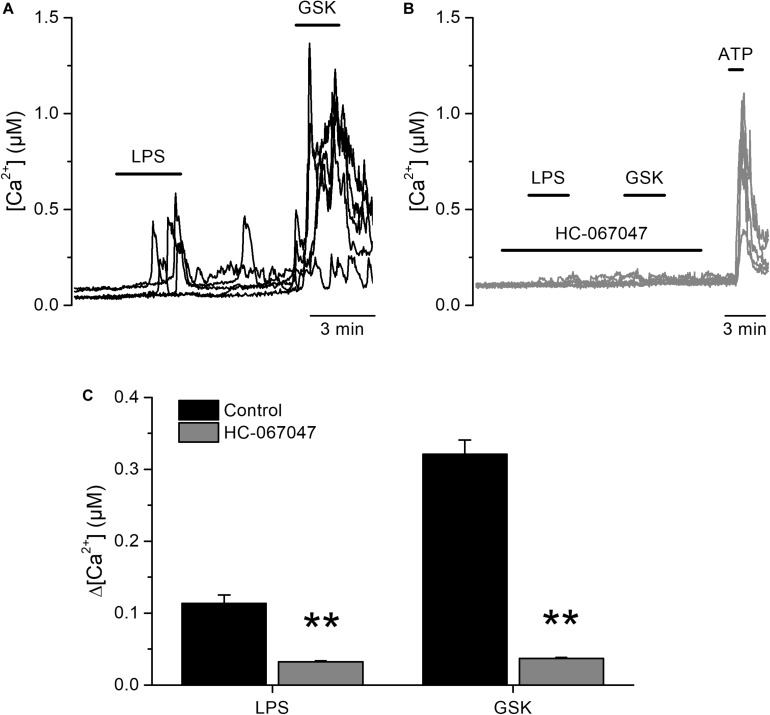
Acute TRPV4-dependent stimulation of human urothelial cells by *E. coli* LPS. **(A,B)** Representative traces of intracellular Ca^2+^ signals recorded in human UCs. LPS (20 μg/ml) and GSK1016790A (GSK, 10 nM) were perfused in control **(A)**, or in the presence of the TRPV4 inhibitor HC-067047 (10 μM) **(B)**. ATP (100 μM) was applied at the end of the latter experiments to assess cellular responsiveness. **(C)** Average amplitudes of intracellular Ca^2+^ responses evoked by LPS (20 μg/ml) or GSK1016790A (GSK, 10 nM) in the absence (*n* = 173) of presence (*n* = 280) of HC-067047 (10 μM). **P < 0.01, Mann–Whitney *U* test.

To determine whether LPS induces acute responses in freshly isolated human urothelial cells (hUCs) we isolated hUCs from bladder strips obtained after cystectomy and followed the dynamics of intracellular Ca^2+^ concentration using Fura-2. We explored the effects of LPS in the micromolar range of concentrations to be able to compare our results to those previously reported (10, 20). We found that acute application of *E. coli* LPS (20 μg/ml) induced a significant increase in the intracellular Ca^2+^ concentration in 41% (72 out of 173) of cells responding to the TRPV4-specific agonist GSK1016790A. The rise in intracellular Ca^2+^ concentration appears rapidly after LPS application and was quickly reversed upon LPS washout (Figure 1A). Pharmacological inhibition of TRPV4 with the specific antagonist HC-067047 (21) strongly suppressed LPS- and GSK1016790A-induced responses (Figures 1B,C), indicating that TRPV4 activation is necessary for the immediate response of hUCs to LPS.

**FIGURE 2:**
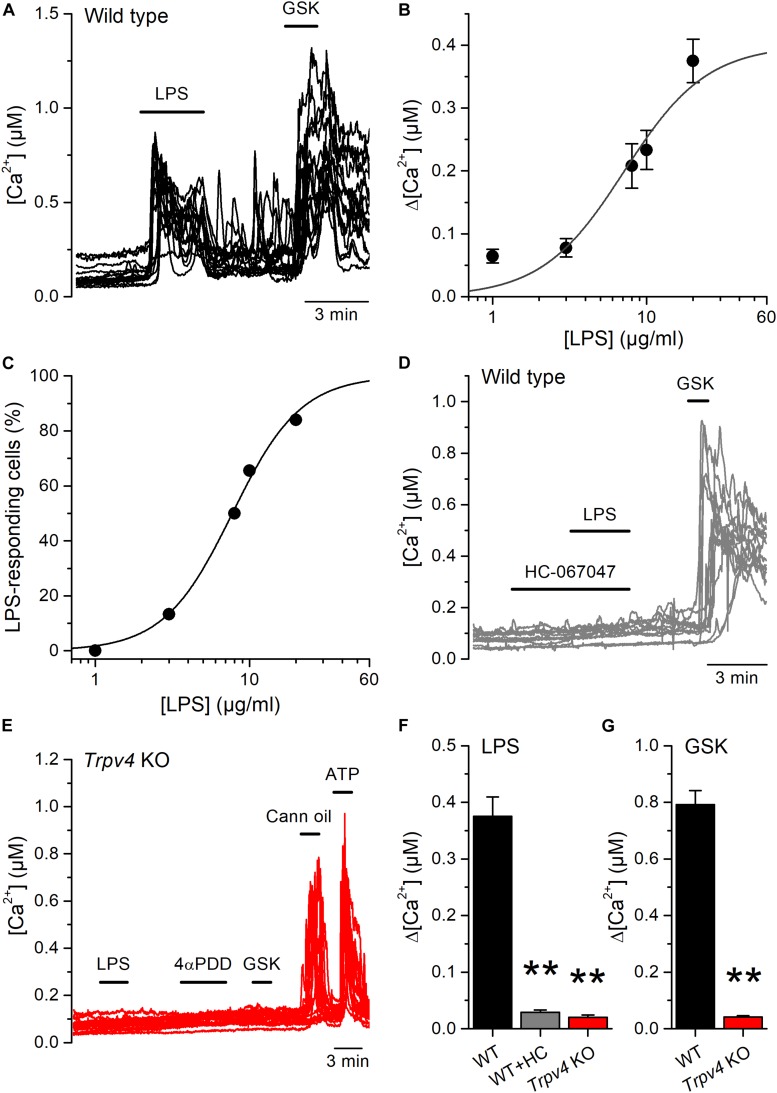
Acute TRPV4-dependent stimulation of mouse urothelial cells by *E. coli* LPS. **(A)** Representative intracellular Ca^2+^ signals recorded in urothelial cells isolated from wild type mice, showing the responses to acute application of LPS (20 μg/ml) or GSK1016790A (GSK, 10 nM). **(B,C)** Concentration dependences of the amplitude (B) and percentage of occurrence **(C)** of LPS-evoked intracellular Ca^2+^ responses in TRPV4-expressing cells (responsive to GSK1016790A 10 nM). The solid lines represent the fits with Hill equations. *n* > 80 cells per data point. **(D)** Representative intracellular Ca^2+^ signals recorded in WT mUCs to which LPS (20 μg/ml) was applied in the presence of the TRPV4 inhibitor HC-067047 (10 μM). GSK1016790A (GSK, 10 nM) was later perfused to assess functional expression of TRPV4. **(E)** Representative intracellular Ca^2+^ signals recorded in urothelial cells isolated from *Trpv4* KO mice. LPS was applied at a concentration of 20 μg/ml. The TRPV4 agonists 4αPDD (2 μM) and GSK1016790A (GSK, 10 nM) were used to evaluate the functional expression of TRPV4. A TRPV2 agonist (cannabis oil, 100 μM) and ATP (10 μM) were used to assess cell responsiveness. **(F,G)** Average amplitude of responses to LPS (20 μg/ml) **(F)** and GSK1016790A (10 nM) **(G)** in urothelial cells isolated from wild type (WT, black bars) and *Trpv4* KO (red bars) mice. HC, HC-067047 (10 μM). The ** symbols indicate *P* < 0.01 with a Dunn’s multiple comparison test.

We next tested the effects of LPS on freshly isolated mouse UC (mUCs), because this preparation was more readily available than human-derived cells, and the possibility to study cells from genetically modified animals. LPS triggered intracellular Ca^2+^ responses in a subpopulation of mUCs derived from wild type (WT) animals. LPS (20 μg/ml) induced a fast and reversible increase in the intracellular Ca^2+^ concentration in 78% (110 out of 141, average amplitude 0.37 ± 0.03 μM) of mUCs responsive to the TRPV4 agonist GSK1016790A (10 nM; Figure 2A). The amplitude of these responses and the proportion of LPS-sensitive cells were concentration-dependent, with effective concentrations (*EC*_50_) of 7.1 ± 1.1 and 7.7 ± 0.3 μg/ml, respectively and Hill coefficients of 1.6 ± 0.4 and 1.9 ± 0.2, respectively (Figures 2B,C). LPS largely failed to increase the intracellular Ca^2+^ concentration in mUCs in the presence of the TRPV4 inhibitor HC-067047 (10 μM) (Figures 2D,F). mUCs harvested from *Trpv4*-deficient animals were also largely insensitive to LPS (Figures 2E,F). As expected, *Trpv4*-deficient mUCs were also unresponsive to the TRPV4 agonists GSK1016790A and 4αPDD (Figures 2E,G). To assess the possibility that the lack of TRPV4 results in a generalized loss of cellular responsiveness, we evaluated the activation of other Ca^2+^-permeable channels expressed in mUCs cells, namely TRPV2 (22) and purinergic receptors (23). Application of the TRPV2 activator cannabis oil or ATP induced acute and reversible increases in cytoplasmic Ca^2+^ concentration (Figure 2E). Altogether, these results demonstrate that, as shown above in hUCs, TRPV4 mediates the LPS-induced acute increase of intracellular Ca^2+^ concentration in mUCs.

**FIGURE 3:**
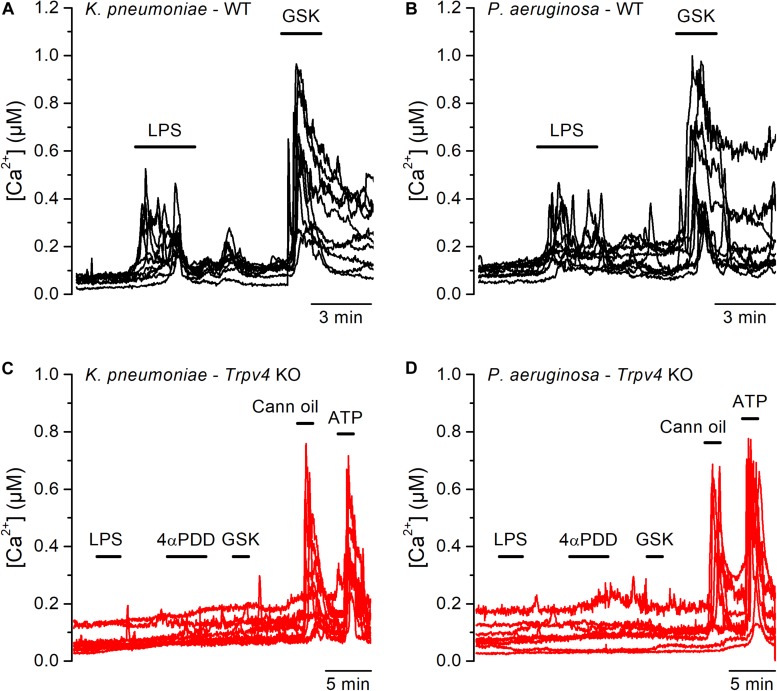
Acute TRPV4-dependent stimulation of mouse urothelial cells by *K. pneumoniae* and *P. aeruginosa* LPS. **(A–D)** Representative intracellular Ca^2+^ signals recorded in urothelial cells isolated from WT (panels **A** and **B**) or *Trpv4* KO (panels **C** and **D**) mice. LPS extracted from *K. pneumoniae* **(A,C)** or *P. aeruginosa* **(B,D)** were applied at a concentration of 20 μg/ml. The TRPV4 agonists 4αPDD (2 μM) and GSK1016790A (GSK, 10 nM) were used to evaluate the functional expression of TRPV4. A TRPV2 agonist (cannabis oil, 100 μM) and ATP (10 μM) were used to assess responsiveness of *Trpv4* KO-derived cells.

Although *E. coli* accounts for 75–90% of UTI, the bacterial spectrum differs in immunocompromised hosts (3). For instance, the opportunistic gram-negative bacteria *Klebsiella pneumoniae* and *Pseudomonas aeruginosa* are common causative agents during nosocomial UTI (2, 24). Thus, we tested whether also LPS from these bacterial strains are capable of activating TRPV4 in mUCs. We found that LPS (20 μg/ml) extracted from *K. pneumoniae* or *P. aeruginosa* induced robust increases in intracellular Ca^2+^ concentration in 80% (72 out of 90 mUCs) and 47% (42 out of 88 mUCs), respectively (Figures 3A,B). Although the amplitudes of the responses elicited by these two LPS were not significantly different from each other (*K. pneumoniae* LPS: 0.14 ± 0.01 μM (*n* = 72), vs. *P. aeruginosa* LPS: 0.12 ± 0.02 μM (*n* = 42); *P* = 0.24, two-tailed unpaired *t* test), they were significantly smaller than the amplitude of the response evoked by *E. coli* LPS (*E. coli*: 0.38 ± 0.03 μM (*n* = 68); *P* < 0.0001, Tukey’s multiple comparison test). LPS from *K. pneumoniae* and *P. aeruginosa* largely failed to increase the intracellular Ca^2+^ concentration in mUCs isolated from *Trpv4* KO mice (Figures 3C,D), confirming that TRPV4 is necessary for acute responses to LPS.

**FIGURE 4:**
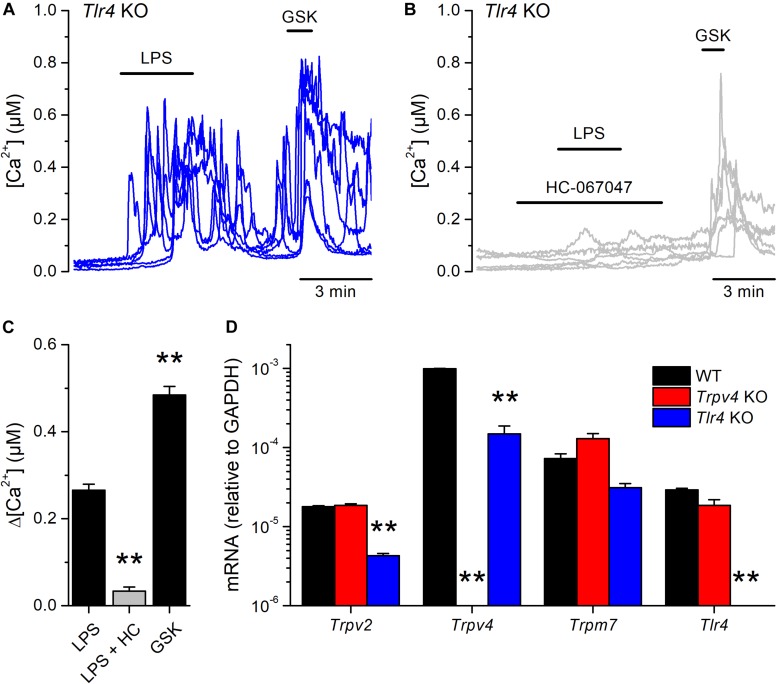
TLR4 is dispensable for the acute response to *E. coli* LPS in mouse urothelial cells. **(A,B)** Representative intracellular Ca^2+^ signals recorded in urothelial cells isolated from *Tlr4* KO mice. LPS (20 μg/ml) was perfused in control **(A)** or in the presence of the TRPV4 inhibitor HC-067047 (10 μM) **(B)**. GSK1016790A (GSK, 10 nM) was applied to assess the functional expression of TRPV4. **(C)** Average amplitude of responses to LPS (20 μg/ml) in control (*n* = 151) or in the presence of HC-067047 (HC, 10 μM) (*n* = 148) in urothelial cells isolated from *Tlr4* KO mice. The average amplitude of the responses to GSK1016790A (GSK, 10 nM) in control (*n* = 151) is also shown for comparison. The ** symbols indicate *P* < 0.01 with a Dunn’s multiple comparison test. **(D)** Expression levels of *Trpv2*, *Trpv4*, *Trpm7* and *Tlr4* mRNA transcripts in urothelial cells derived from WT, *Trpv4* KO and *Tlr4* KO mice. The bars represent mean ± SEM, (*n* = 3). The ** symbols indicate *P* < 0.01 with a Tukey’s multiple comparison test.

Next, we tested whether the canonical immune receptor TLR4 contributes to the acute responses of mUCs to LPS. We found that 65% (99 out of 151) of the mUCs isolated from *Tlr4* KO mice exhibited an increase in intracellular Ca^2+^ concentration upon LPS application (Figures 4A,C). As found in WT cells, both the amplitude of the LPS-induced intracellular Ca^2+^ responses (Figures 4B,C) and the percentage of LPS-responding cells (1.3%; 2 out of 148) were significantly smaller in the presence of the TRPV4 inhibitor HC-067047, compared to untreated *Tlr4* KO mUCs (*P* < 0.001, Dunn’s multiple comparison test and *P* < 0.0001, Fisher’s exact test, respectively). Interestingly, *Tlr4*-deficient mUCs exhibited a lower fraction of responding cells (65%) and smaller amplitude of responses to LPS (0.27 ± 0.01 μM) in comparison to WT mUCs (*P* = 0.019, Fisher’s exact test and *P* = 0.0008, two-tailed unpaired *t* test, respectively). Furthermore, we found that also the responses to GSK1016790A were significantly smaller in *Tlr4*-deficient cells (WT: 0.79 ± 0.05 μM vs. *Tlr4* KO*:* 0.49 ± 0.02 μM; *P* < 0.0001, two-tailed unpaired *t* test). These weaker TRPV4-mediated responses in *Tlr4* KO mUCs may be related to the lower expression levels of TRPV4, since comparative transcripts analysis showed significant lower expression of *Trpv4* (and *Trpv2*) in *Tlr4* KO than in WT mUCs (Figure 4D). Altogether, these findings confirm that TRPV4 is the main contributor to the fast increase in intracellular Ca^2+^ concentration induced by LPS in mUCs and demonstrate that the TLR4 signaling pathway is not critically required for LPS-induced activation of TRPV4.

### LPS-Induced Inflammatory Gene Expression Is Enhanced in Trpv4-Deficient Urothelial Cells

**FIGURE 5:**
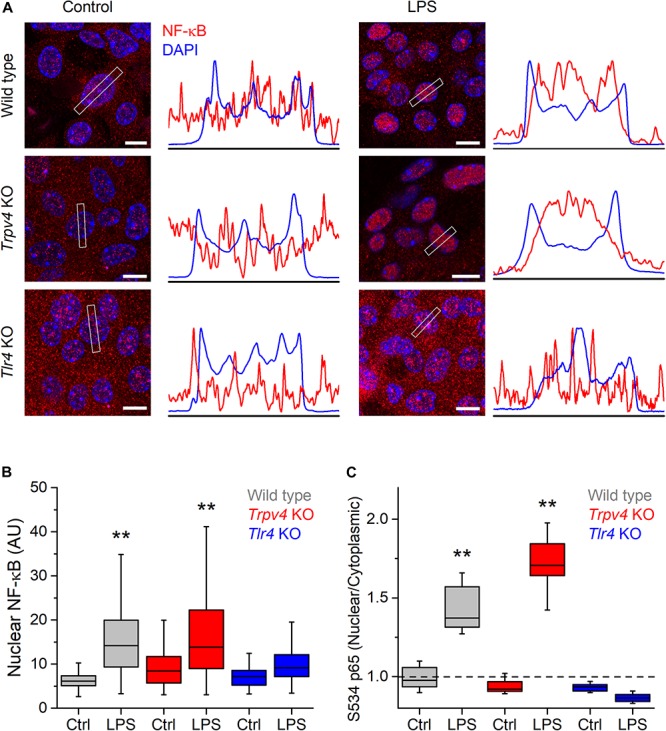
TRPV4 is not required for the phosphorylation and nuclear translocation of NF-κB induced by *E. coli* LPS in mouse urothelial cells. **(A)** Representative confocal immunofluorescence microscopy images of fixed mUCs in control or 30 min after treatment with LPS (20 μg/ml). Cells were stained with NF-κB p65 (red) and DAPI nuclear staining (blue). Scale bar, 10 μm. The average linear intensity along the gray rectangle is represented next to each image. **(B)** Mean intensity of nuclear NF-κB p65 staining in mUCs in control or 30 min after treatment with LPS (20 μg/ml, 30 min). The data is represented in a box plot indicating the median (black line), the 25–75 percentiles (box) and the 10–90 percentile range (whiskers). The ** symbols indicate *P* < 0.01 and with a Tukey’s multiple comparison test (the *n* values (control/LPS) are 53/100 for WT, 96/92 for *Trpv4* KO and 88/103 for *Tlr4* KO). **(C)** Average nuclear/cytoplasmic ratio of S534 p65 subunit in mUCs in control or 30 min after treatment with LPS (20 μg/ml, 30 min). The data is represented in a box plot indicating the median (black line), the 25–75 percentiles (box) and the 10–90 percentile range (whiskers). The ** symbols indicate *P* < 0.01, with a Tukey’s multiple comparison test. At least eight randomly selected images were analyzed per conditions from three independent experiments.

Lipopolysaccharides triggers an increase in intracellular Ca^2+^ concentration that leads to Ca^2+^-dependent phosphorylation of NF-κB, inducing its nuclear translocation and a consequent increased production of proinflammatory cytokines in a line of human carcinoma bladder epithelial cells and in macrophages (10, 25). Thus, we sought to determine whether the TRPV4-mediated increase in intracellular Ca^2+^ induced by LPS triggers NF-κB translocation in mUCs. For this, we incubated these cells with LPS during 30 min and quantified the presence of NF-κB in the nucleus. Untreated WT mUCs exhibited a scattered distribution of NF-κB, with similar expression in the nucleus and the cytoplasm, whereas LPS-treated cells displayed a significant increase in nuclear NF-κB and S534-phosphorylated p65 subunit (Figures 5A–C and Supplementary Figures 1, 2). *Trpv4*-deficient mUCs showed results similar to WT cells, but *Tlr4* KO mUCs appeared unresponsive to LPS (Figures 5A–C and Supplementary Figures 1, 2).

**FIGURE 6:**
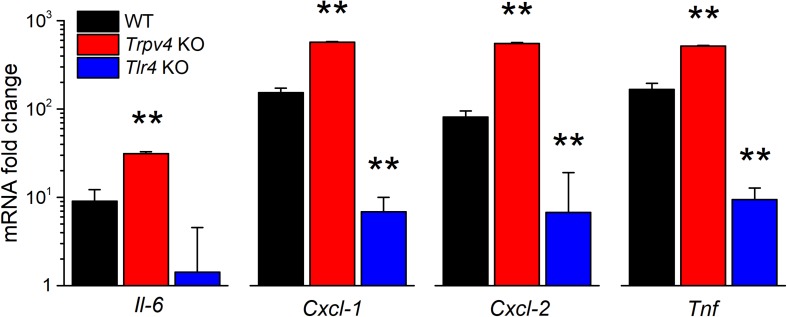
Differential regulation of LPS-induced cytokine gene expression by TRPV4 and TLR4 in mUCs. Fold change of the expression levels of pro-inflammatory genes in urothelial cells derived from WT, *Trpv4* KO and *Tlr4* KO mice and treated with *E. coli* LPS (20 μg/ml, 30 min). The data is presented as mean ± SEM. of the expression levels relative to the respective values obtained in the untreated condition (*n* = 3). The ** symbols indicate *P* < 0.01, with a Tukey’s multiple comparison test.

We also tested for the implication of TRPV4 in the regulation of the expression of pro-inflammatory cytokines. We found that the LPS challenge induced a significantly larger increase in *Il-6*, *Cxcl-1*, *Cxcl-2*, and *Tnf* transcript levels in TRPV4-deficient mUC than in WT mUC (Figure 6). Interestingly, LPS-treated *Tlr4* KO mUC did not display *Il-6* upregulation, but did show about 10-fold increased expression of *Cxcl-1*, *Cxcl-2*, and *Tnf* (Figure 6).

### TRPV4 Is Required for LPS-Induced Increase in Bladder Voiding Frequency

Finally, we determined whether intravesical infusion of LPS produces acute changes in the mouse bladder voiding reflex using cystometry *in vivo* (19). Given that ablation of TRPV4 induces a reduction of the voiding frequency in mice (21), we hypothesized that LPS-induced activation of the channel may produce the opposite effect.

**FIGURE 7:**
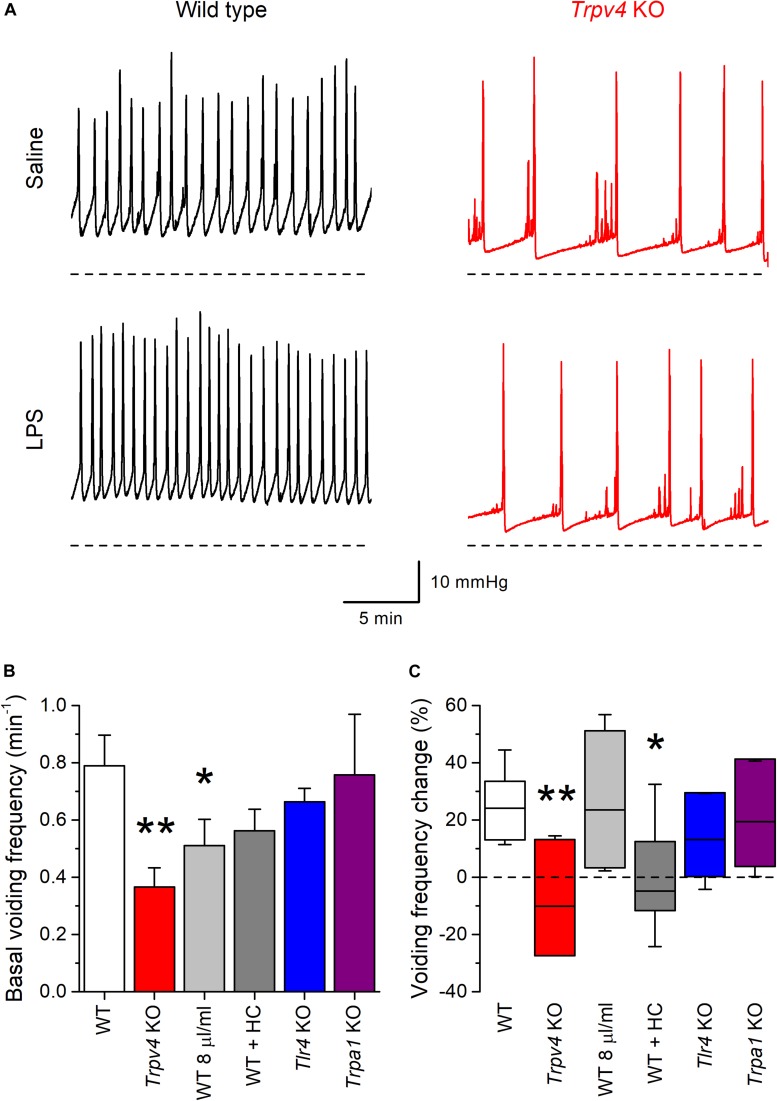
TRPV4 mediates the increase of mouse bladder voiding frequency induced by intravesical instillation of *E. coli* LPS. **(A)** Traces of intravesical pressure recordings performed in wild type (black) and *Trpv4* KO (red) mice, during infusion of control saline (NaCl 0.9%) or *E. coli* LPS (200 μg/ml). **(B)** Basal voiding frequency of WT (*n* = 14), *Trpv4* KO (*n* = 9), WT perfused at a slow rate of 8 μl/min (*n* = 8), WT administered the TRPV4 inhibitor HC-067047 (HC, 2.35 mg/kg, *n* = 6), *Tlr4* KO (*n* = 9) and *Trpa1* KO (*n* = 7) mice. The data is presented as mean ± SEM. The * and ** symbols indicate *P* < 0.05 and *P* < 0.01, respectively with a Mann–Whitney *U* test vs. WT. **(C)** Change in the voiding frequency determined over 20 min after the first 10 min of intravesical instillation of *E. coli* LPS (200 μg/ml). The data is represented as the median (black line), the 25–75 percentiles (box) and the 95% confidence interval (whiskers). The * and ** symbols indicate *P* < 0.05 and *P* < 0.01, respectively with a Mann–Whitney *U* test vs. WT. WT (*n* = 13), *Trpv4* KO (*n* = 9), WT perfused at a slow rate of 8 μl/min (*n* = 7), WT administered the TRPV4 inhibitor HC-067047 (HC, 2.35 mg/kg, *n* = 6), *Tlr4* KO (*n* = 9), and *Trpa1* KO (*n* = 7).

During infusion of saline solution at a rate of 20 μl/min *Trpv4* KO mice displayed a basal voiding frequency that was significantly lower than that of WT animals (Figures 7A,B; *P* = 0.0011), as previously reported (13, 26). Acute intravesical administration of LPS (200 μg/ml in saline) via the infusion catheter induced an increase in the voiding frequency in WT (*P* = 8 × 10^–4^), but not in *Trpv4*-deficient mice (*P* = 0.74) (Figures 7A,C).

The lack of effect of LPS in the latter could be related to the lower rate of administration of this compound to the bladder, given the lower basal voiding frequency in these animals. To assess this possibility, we performed another series of experiments in WT mice using an intravesical infusion rate 8 μl/min to adjust the basal voiding frequency in these animals to values closer to that of *Trpv4* KO mice (Figure 7B). In this condition, LPS increased the voiding frequency to levels similar to those found in WT animals at a perfusion rate of 20 μl/min (*P* = 0.48; Figure 7C). WT mice under pharmacological inhibition of TRPV4 by intraperitoneal administration of HC-067047 (2.35 mg/kg) displayed a basal voiding frequency that was not different from that of control animals (Figure 7B; *P* = 0.18). However, instillation of LPS did not result in changes in voiding frequency in HC-067047-treated mice (*P* = 0.58; Figure 7C), which is consistent with the result obtained in *Trpv4* KO animals.

We also tested the implication of TLR4 in the voiding response by performing experiments in *Tlr4* KO mice. These animals displayed a basal voiding frequency and response to LPS that were not different from those of WT mice (Figures 7B,C). Finally, given its reported role in acute inflammation and pain responses to LPS (27), we assessed the implication of TRPA1. Mice deficient of this channel showed no difference in basal voiding frequency or response to LPS with respect to WT animals (Figures 7B,C).

## Discussion

The quick defensive responses of cells lining the urinary tract and bladder wall are determinant in limiting further bacterial colonization and tissue damage. Of particular interest in this regard are the fast increases in intracellular Ca^2+^ concentration and in proinflammatory gene expression induced by acute LPS application that were reported in a carcinoma human bladder epithelium cell line (10). In this study we found that fast LPS-induced Ca^2+^ responses are present also in primary cultured UCs isolated from human and mouse bladder samples, and obtained pharmacologic and genetic evidence indicating that TRPV4 activation is a necessary factor for this effect in both cell types.

Of note, we found that mUCs express mRNA of TRPV2 and TRPM7, two channels that were reported to mediate Ca^2+^ influx required for TLR4-dependent cytokine production in macrophages (25, 28). However, these channels do not play a critical role in the generation of the fast Ca^2+^ responses to LPS in mUCs, because these cells fail to respond when TRPV4 function is ablated. The activation of TRPM7 by LPS in macrophages occurs through interaction with the adaptor protein CD14, which facilitates the inclusion of this channel in the TLR4-MD2-CD14 complex (25). This mechanism is unlikely to operate in UCs since they lack expression of CD14 (29). Regarding TRPV2, we have previously reported that this channel is larger insensitive to LPS (30), and therefore is not expected to contribute to LPS-induced Ca^2+^ responses. Importantly, our data in *Tlr4* KO mUCs demonstrate that this receptor is not critical for LPS-induced activation of TRPV4. These conclusions fall in line with our previous observations in airway epithelial cells (12), suggesting for a general role of TRPV4 as LPS effector in epithelial cells.

Another feature that recapitulates what was found in airway epithelial cells (12) is that LPS elicits intracellular Ca^2+^ responses only in a fraction of UCs responding to the potent TRPV4 agonist GSK1016790A. This is probably due to the fact that LPS is a weaker channel agonist (31). The apparent effective stimulatory concentration of LPS on TRPV4 in mUCs we report here (∼7 μg/ml) is very similar to that previously found in airway epithelial cells (11 μg/ml) (12). These values are in the same range as that reported for the broadly tuned chemosensory channel TRPA1 (32) (∼3 μg/ml) (27), and lower than for the other sensory TRP channels, TRPV1, TRPV2, TRPM3, and TRPM8 (30). This confirms TRPA1 and TRPV4 as the most sensitive to LPS of the sensory TRP channels studied so far in this respect.

The effect of endotoxins on intracellular Ca^2+^ levels in mUCs and the implication of TRPV4 were confirmed for LPS extracted from *K. pneumoniae* and *P. aeruginosa*, which are two of the most frequent bacterial strains associated with the presence of indwelling urinary catheters (33). The weaker action of LPS from these strains compared to that of *E. coli*-derived LPS in activating TRPV4 is reminiscent of the stronger agonist effect of *E. coli* in TRPA1 (27). The molecular mechanism underlying TRPV4 activation by LPS remains to be fully clarified. However, because of the widely reported mechanosensitivity of this channel (34–36), we hypothesize that it could be similar as that operating in TRPA1, i.e., via the detection of LPS-induced mechanical perturbations in the plasma membrane (27, 37–39).

Our results show that TRPV4 activation does not influence the level of TLR4-dependent phosphorylation of NF-κB nor its translocation to the nucleus observed after 30 min stimulation with LPS. On the other hand, TRPV4-deficient cells displayed enhanced LPS-induced gene expression of the proinflammatory cytokines *Il-6*, *Cxcl-1*, *Cxcl-2*, *Tnf*, irrespective of whether this depended fully or not on TLR4 activation. The fact that this was also observed for *Il-6* and *Cxcl-1*, although not for *Cxcl-2*, in mouse tracheobronchial cells, suggests that the increase in Ca^2+^/Na^+^ and/or the depolarization resulting from TRPV4 activation can have a negative regulatory action on LPS-induced gene expression of some proinflammatory cytokines in epithelial cells. The elucidation of the mechanism underlying this regulation requires further investigation.

The effects of luminal LPS in the urinary bladder are not restricted to long-term histological inflammatory changes in the bladder wall, but also to an immediate increase in voiding frequency (11). Our cystometry experiments demonstrate that TRPV4 is a key mediator of the LPS-induced acute increase in voiding frequency. Notably, these effects are already established within minutes of LPS installation, a time-frame in which histological inflammatory changes are not seen yet (6). The rapidity of these events is likely linked to the activation of sensory fibers densely innervating the bladder wall. However, the full abrogation of changes in voiding frequency in *Trpv4* KO mice excludes the direct interaction of LPS with TRPA1 (27) in sensory afferents as the trigger for changes in voiding pattern. Instead, TRPV4 activation may lead to the release of H_2_O_2_ (40), a known activator of TRPA1 (41, 42). However, the lack of difference in the voiding response to intravesical LPS administration between *Trpa1* KO and WT animals indicates that TRPA1 channels are not implicated. Alternatively, the activation of TRPV4 in UCs by LPS may induce the release of ATP (13, 36, 43), triggering afferent pathways through activation of purinergic receptors (13).

Altogether, our results suggest that UCs can recognize bacterial colonization through TRPV4 activation by LPS. The increase in voiding frequency induced by LPS may explain the strong urge to void frequently during cystitis, even immediately after the bladder is emptied. Together with UCs exfoliation during cystitis (44, 45), the increased flushing activity mediated by TRPV4 may contribute to the elimination of the pathogen from the bladder lumen.

In more general terms, our present findings further support the notion that TRP channels may function as sensors of bacterial endotoxins, playing crucial roles in the timely detection of invading gram-negative bacteria (38). Importantly, they indicate that the pharmacological inhibition of TRPV4, which has been suggested for the treatment of several airway diseases (46–48), as well as of cyclophosphamide-induced cystitis (21), may lead to undesirably decreased defensive responses of the bladder against rather common bacterial infections.

## Data Availability Statement

The datasets generated for this study are available on request to the first and corresponding authors.

## Ethics Statement

The animal study was reviewed and approved by the KU Leuven Ethics Committee.

## Author Contributions

YA and KT conceived and designed the project, and wrote the manuscript. JF and YA isolated and cultured the human urothelial cells. YA conducted and analyzed the Ca^2+^ imaging experiments. RN and YA performed the confocal imaging and quantitative RT-PCR. PU, SP, and YA designed and conducted the cystometry recordings. AS contributed to the data analyses. DD, TG, WE, and TV contributed to the interpretation of data. All authors edited the manuscript.

## Conflict of Interest

The authors declare that the research was conducted in the absence of any commercial or financial relationships that could be construed as a potential conflict of interest.

## Supplementary Material

FIGURE S1 TRPV4 is not required for the LPS-induced nuclear translocation of the p65 subunit of NF-κB in mouse urothelial cells. Single-color confocal images of NF-κB p65 (red) and DAPI nuclear staining (blue). The corresponding merged images are shown in Figure 5A. Scale bar, 10 μm.

FIGURE S2 TRPV4 is not required for the LPS-induced phosphorylation of S534 p65 subunit of NF-κB in mouse urothelial cells. Representative confocal immunofluorescence microscopy images of fixed mUCs in control or 30 min after treatment with LPS (20 μg/ml). Cells were stained with phospho p65 NF-κB (red) and DAPI nuclear staining (blue). Scale bar, 10 μm. The histograms show the corresponding pixel intensity distribution (8-bit scale) of phospho p65 NF-κB staining in the cytoplasmic area (gray-shaded bars) or within the nuclear area (blue bars). Ratios represented in Figure 5C were calculated using the mean intensity values from the pixel intensity distribution in these two areas.
