# Supplementary material for: TRPV4 Mediates Acute Bladder Responses to Bacterial Lipopolysaccharides
Source: Front Immunol. 2020 May 6;11:799. doi: 10.3389/fimmu.2020.00799 (PMC7218059; doi:10.3389/fimmu.2020.00799)
Supplement: FIGURE S1 — TRPV4 is not required for the LPS-induced nuclear translocation of the p65 subunit of NF-κB in mouse urothelial cells. Single-color confocal images of NF-κB p65 (red) and DAPI nuclear staining (blue). The corresponding merged images are shown in Figure 5A. Scale bar, 10 μm. [file Image_1.pdf]

Supplementary Figure S1

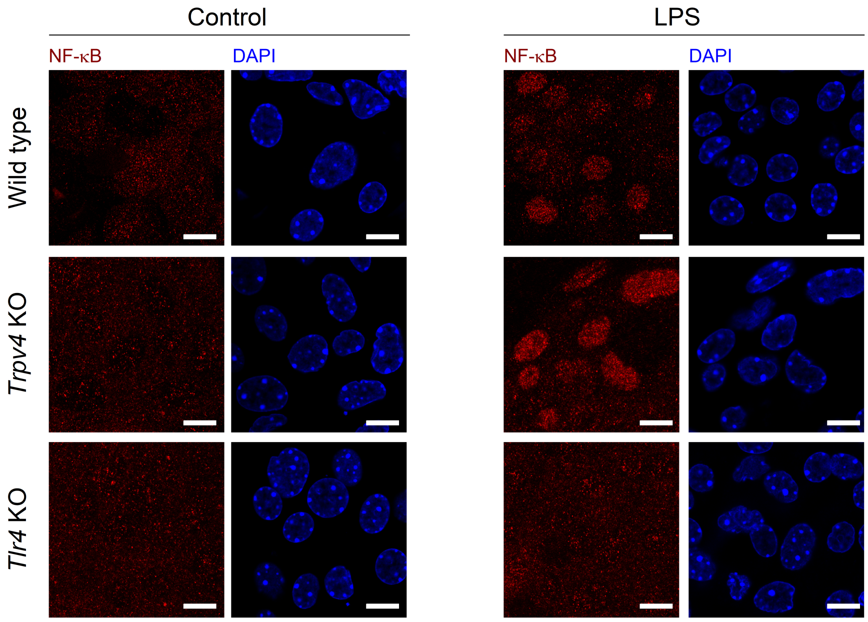

Supplementary Figure S1. TRPV4 is not required for the LPS-induced nuclear translocation of the p65 subunit of NF-κB in mouse urothelial cells. Single-color confocal images of NF-κB p65 (red) and DAPI nuclear staining (blue). The corresponding merged images are shown in Figure 5A. Scale bar, 10 μm.
