# Supplementary material for: TRPV4 Mediates Acute Bladder Responses to Bacterial Lipopolysaccharides
Source: Front Immunol. 2020 May 6;11:799. doi: 10.3389/fimmu.2020.00799 (PMC7218059; doi:10.3389/fimmu.2020.00799)
Supplement: FIGURE S2 — TRPV4 is not required for the LPS-induced phosphorylation of S534 p65 subunit of NF-κB in mouse urothelial cells. Representative confocal immunofluorescence microscopy images of fixed mUCs in control or 30 min after treatment with LPS (20 μg/ml). Cells were stained with phospho p65 NF-κB (red) and DAPI nuclear staining (blue). Scale bar, 10 μm. The histograms show the corresponding pixel intensity distribution (8-bit scale) of phospho p65 NF-κB staining in the cytoplasmic area (gray-shaded bars) or within the nuclear area (blue bars). Ratios represented in Figure 5C were calculated using the mean intensity values from the pixel intensity distribution in these two areas. [file Image_2.pdf]

Supplementary Figure S2

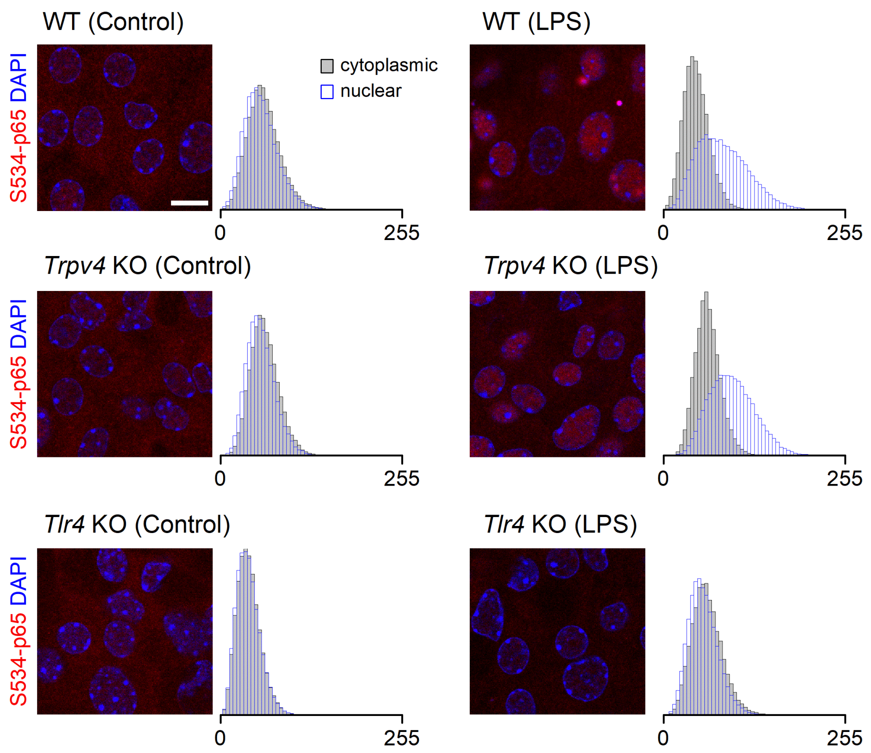

Supplementary Figure S2. TRPV4 is not required for the LPS-induced phosphorylation of S534 p65 subunit of NF-κB in mouse urothelial cells. Representative confocal immunofluorescence microscopy images of fixed mUCs in control or 30 min after treatment with LPS (20 µg/ml). Cells were stained with phospho p65 NF-κB (red) and DAPI nuclear staining (blue). Scale bar, 10 µm. The histograms show the corresponding pixel intensity distribution (8-bit scale) of phospho p65 NF-κB staining in the cytoplasmic area (gray-shaded bars) or within the nuclear area (blue bars). Ratios represented in Figure 5C were calculated using the mean intensity values from the pixel intensity distribution in these two areas.
